# Global Distribution and Evolutionary History of Enterovirus D68, with Emphasis on the 2014 Outbreak in Ontario, Canada

**DOI:** 10.3389/fmicb.2017.00257

**Published:** 2017-03-01

**Authors:** Alireza Eshaghi, Venkata R. Duvvuri, Sandra Isabel, Philip Banh, Aimin Li, Adriana Peci, Samir N. Patel, Jonathan B. Gubbay

**Affiliations:** ^1^Department of Clinical Laboratory and Microbiology Sciences, Public Health Ontario, TorontoON, Canada; ^2^Department of Paediatrics, The Hospital for Sick Children, University of Toronto, TorontoON, Canada; ^3^Department of Laboratory Medicine and Pathobiology, University of Toronto, TorontoON, Canada; ^4^Department of Microbiology, Mount Sinai Hospital, TorontoON, Canada

**Keywords:** enterovirus D68, phylogenetic analysis, whole genome sequencing, cluster lineage, next-generation sequencing, outbreak

## Abstract

Despite its first appearance in 1962, human enterovirus D68 (EV-D68) has been recognized as an emerging respiratory pathogen in the last decade when it caused outbreaks and clusters in several countries including Japan, the Philippines, and the Netherlands. The most recent and largest outbreak of EV-D68 associated with severe respiratory illness took place in North America between August 2014 and January 2015. Between September 1 and October 31 2014, EV-D68 infection was laboratory confirmed among 153/907 (16.9%) persons tested for the virus in Ontario, Canada, using real time RT-PCR and subsequent genotyping by sequencing of partial VP1 gene. In order to understand the evolutionary history of the 2014 North American EV-D68 outbreak, we conducted phylogenetic and phylodynamic analyses using available partial VP1 genes (*n* = 469) and NCBI available whole genome sequences (WGS) (*n* = 38). The global EV-D68 phylogenetic tree (*n* = 469) reconfirms the divergence of three distinct clades A, B, and C from the prototype EV-D68 Fermon strain as previously documented. Two sub-clades (B1 and B2) were identified, with most 2014 EV-D68 outbreak strains belonging to sub-cluster B2b2 (one of the two emerging clusters within sub-clade B2), with two signature substitutions T650A and M700V in BC and DE loops of VP1 gene, respectively. The close homology between WGS of strains from Ontario (*n* = 2) and USA (*n* = 21) in the recent EV-D68 outbreak suggests genetic relatedness and also a common source for the outbreak. The time of most recent common ancestor of EV-D68 and the 2014 EV-D68 outbreak strain suggest that the viruses possibly emerged during 1960–1961 and 2012–2013, respectively. We observed lower mean evolutionary rates of global EV-D68 using WGS data than estimated with partial VP1 gene sequences. Based on WGS data, the estimated mean rate of evolution of the EV-D68 B2b cluster was 9.75 × 10^-3^ substitutions/site/year (95% BCI 4.11 × 10^-3^ to 16 × 10^-3^).

## Introduction

Enterovirus D68 (EV-D68) is a non-enveloped, positive-sense single strand RNA virus belonging to species enterovirus D of the *Enterovirus* genus within the *Picornaviridae* family. Following its first isolation in 1962 from pediatric patients with acute respiratory symptoms in the United States of America (USA), EV-D68 has been detected with increasing frequency in respiratory tract specimens (Schieble et al., 1967). Among *Enterovirus* genus members, EV-D68 is the only serotype with characteristics commonly found in rhinoviruses such as acid lability and better growth at lower temperatures(33°C) (Blomqvist et al., 2002). Infection with EV-D68 can cause a wide range of clinical presentations, from mild acute respiratory illness (ARI), including influenza-like symptoms, to severe acute lower respiratory tract infections such as pneumonia, bronchiolitis and rarely death (Centers for Disease Control and Prevention (CDC), 2011; Kreuter et al., 2011). Recent studies suggested a possible aetiological role of EV-D68 in meningitis and acute flaccid paralysis (AFP) (Lang et al., 2014). Since 2006, clusters or outbreaks of ARI due to EV-D68 have been documented worldwide in: China (2006 and 2008), the Philippines (2008–2009), France (2009–2010), Japan (2010), USA (2009 and 2014), Netherlands (1994–2010), Italy (2008–2014), Thailand (2006–2011), and South Africa (2000–2001) (European Centre for Disease Prevention and Control (ECDC), 2014; Piralla et al., 2014). Compared to the small number of EV-D68 strains detected from clinical specimens prior to 2006 (*n* = 26 from 1970 to 2005 in the USA), the increasing numbers of global outbreaks since 2008 indicate the importance of EV-D68 as an emerging respiratory pathogen (Rahamat-Langendoen et al., 2011; Tokarz et al., 2011; Meijer et al., 2012).

Between 1991 and 2013, only 82 isolates of EV-D68 were reported by Public Health Agency of Canada-National Microbiology Laboratory (PHAC-NML) (Booth et al., 2015; Edwin et al., 2015). A study from Hamilton, ON, Canada conducted during September to October of 2010 and 2011, detected EV-D68 in 7% (7/92) and 1% (4/400) of self-collected nasal swabs from university students with and without acute respiratory symptoms, respectively (Granados et al., 2015). The recent USA outbreak of EV-D68 started in August 2014, and soon after reached Canada. The US Centers for Disease Control and Prevention (CDC) reported 1,153 laboratory confirmed EV-D68 cases in 49 states from mid-August 2014 to January 2015 (European Centre for Disease Prevention and Control (ECDC), 2014). This recent outbreak was the first experience of such a large EV-D68 outbreak in Canada. From August to December 2014, active sentinel and enhanced passive surveillance approaches were conducted in Canada to assess the risk of 2014 EV-D68 infection along with its outbreak features (Skowronski et al., 2015). In Canada, 268 pediatric hospitalized cases were reported between September 1 and 30, 2014 from three provinces: Ontario (*n* = 210), Alberta (*n* = 45), and British Columbia (*n* = 13) (Edwin et al., 2015). In Ontario, EV-D68 was detected in 16.9% (*n* = 153/907) of upper respiratory tract samples submitted to Public Health Ontario Laboratories (PHOL) for testing between September 1 and October 31, 2014. More than 50% of cases were children below 5 years of age (median age 3 years) with most (684/907; 75.6%) testing performed on children less than 10 years of age. Cases were not more likely to be hospitalized than controls, and were more likely to be identified in September than October (OR 8.07; 95% CI 5.15 to 12.64). There was no difference in hospitalization status between cases and controls (Peci et al., 2015).

The EV-D68 genome (approximately 7.5 kb in length) contains a 5′ untranslated region (UTR) followed by a single open reading frame and a short flanking 3′UTR non-coding region. The polyprotein is processed by viral proteases, resulting in four structural (VP4, VP2, VP3, and VP1) and seven non-structural (2A, 2B, 2C, 3A, 3B, 3C, and 3D) proteins (Pallansch and Roos, 2007). Among the structural proteins, VP1 is considered an important genotype determinant due to the presence of two loop structures, BC and DE, on the viral surface. Their association with enterovirus antigenic epitopes was previously reported (Norder et al., 2003). Recent phylogenetic analysis of VP1 nucleotide sequences indicates that three genetic groups, designated as lineages 1, 2, and 3, or clades A, B, and C, are circulating worldwide (Linsuwanon et al., 2012; Meijer et al., 2012; Tokarz et al., 2012).

In this report, we studied the genetic diversity and evolutionary history of EV-D68, with emphasis on the 2014 outbreak in Ontario. We carried out genotype classification of EV-D68, lineage or clade global distribution, time of the most recent common ancestor (tMRCA), and rate of evolution using phylogenetic and phylodynamic analyses using VP1 partial gene sequences and whole genome sequences (WGS) from Ontario, and worldwide sequences available in GenBank as of December 16, 2014. Furthermore, to determine presence of genomic markers associated with this outbreak and their possible association with rapid spread of EV-D68, the complete genome of two representatives of EV-D68 strains from Ontario were sequenced from clinical specimens using next-generation sequencing (NGS) and compared to publicly available genomes.

## Materials and Methods

### Specimen Collection and Sequencing

Specimens submitted to PHOL for EV-D68 testing were collected from patients who presented with respiratory symptoms in different health care settings across Ontario. Early in the outbreak, testing was performed at the PHAC-NML, and as of October 8, 2014, testing was implemented at PHOL. Detection of EV-D68 was performed by pan-enterovirus real-time reverse transcription PCR (rRT-PCR) targeting 5′UTR followed by partial VP1 gene sequencing to determine the molecular serotype as previously described (Nix et al., 2006; Oberste et al., 2013). Thirty-four EV-D68-positive specimens collected during the 2014 Ontario EV-D68 outbreak, which started in September 2014, were included in this study. Furthermore, to study the genome characteristics of the 2014 EV-D68 outbreak in Ontario, two primary specimens (NCBI accession numbers KT835407 and KT835408), representing the two main genetic clusters based on sequence analysis of VP1 region, were selected for whole genome sequencing.

For WGS, the extracted total nucleic acid from nasopharyngeal swabs (NPS) was subjected to removal of human rRNA using the NEBNext rRNA Depletion Kit (New England Biolabs, Ipswich, MA, USA) (Morlan et al., 2012; Adiconis et al., 2013). The DNA library was prepared using the TruSeq RNA library preparation kit v2, normalized, and run on the Illumina Miseq (Illumina, San Diego, CA, USA) using the 2 × 150 bp paired-end reads option. Analysis of the metagenomic data and identification of EV-D68 sequences were performed using the SURPI (Sequence-based Ultra-Rapid Pathogen Identification) pipeline and reference alignment using bowtie2 (v2.2.5) (Langmead and Salzberg, 2012; Naccache et al., 2014). Reference sequences were downloaded from NCBI and SURPI’s curated databases. A consensus sequence was also generated for each sample using the command “samtoolsmpileup -ufref.faaln.bam | bcftools view -cg - | vcfutils.pl vcf2fq >cns.fq” (Li et al., 2009; Li, 2011).

### Sequence Data Set Construction

In our study, we used three discrete datasets of EV-D68 for evolutionary analysis. These were named (1): WGS, containing strains from clade B with available WGS in GenBank, between 1999 and 2014, and the Fermon reference strain (AY426531.1) from 1967 (*n* = 38, two from Ontario, 21 from USA, and 15 from Other countries), (2): VP1-All includes all EV-D68 (*n* = 469, 34 from Ontario, and 435 from global), (3): VP1-B2b (*n* = 74), only contains strains belonging to sub-clade B2b, including the strains collected globally during the 2014 outbreak (Supplementary Table S1).

As noted above, VP1-All consisted of 34 partial VP1 sequences from the recent EV-D68 outbreak in Ontario and 435 EV-D68 VP1 gene partial sequences (339 bp of the hypervariable region corresponding to nt positions 2521–2859 of the Fermon reference strain AY426531.1) containing exact date of collection (from 1962 to 2014) retrieved from NCBI’s GenBank sequence database. Although EV-D68 cases had been reported in Canada prior to the 2014 outbreak, we did not find any submissions of Canadian EV-D68 VP1 gene sequences in GenBank from prior to 2014. Therefore, it is difficult to describe specific clades previously circulating in Ontario.

### Evolutionary Analyses

Phylogenetic analysis was carried out using the 469 partial VP1 EV-D68 nucleotide sequences (339 bp) using the Neighbor-Joining method. Evolutionary distances were computed using Maximum Composite Likelihood model between sequences implemented in MEGA v6.06 (Tamura et al., 2013). A Bayesian Markov chain Monte Carlo (MCMC) method implemented in BEAST v1.8.0 (Drummond and Rambaut, 2007) was used to construct Maximum Clade Credibility (MCC) trees and estimate temporal phylogenies and rates of evolution. Both strict and relaxed clocks (uncorrelated relaxed clock) were employed with constant population size and Bayesian Skyline plot (BSP) coalescent models to compare homogenous and heterogeneous substitution rates across phylogenetic branches, respectively (Drummond et al., 2006). The (continuous) gamma model with substitution model HKY85 was used to estimate evolution. The BEAST generated log and tree files were combined and model convergence was ensured by confirming that the effective sampling size (ESS) of all parameters was ≥200 by using Tracer v1.6 (Rambaut et al., 2014). The uncertainty in the estimates is indicated by 95% Bayesian Credible Interval (BCI) values. Tree Annotator v1.8.0 program (included in BEAST) was used to summarize the information in a sample of trees by choosing the tree with the maximum product of posterior probabilities. The Bayesian MCC phylogeny annotated with divergence time, lineages, and evolutionary rate summaries was used as a representation of the evolutionary history of EV-68 and phylogeny visualized using FigTree v1.4.2^1^. Null (i.e., the one with the lower marginal likelihood) and alternative models were compared for their fit to the data using Bayes factors based on the harmonic mean estimator as calculated by the program Tracer v.1.4 from the BEAST package, where 2 > Bayes factor ≤ 6 indicates positive evidence against the null model; >6 > Bayes factor ≤ 10 indicates strong evidence against the null model; and Bayes factor > 10 indicates very strong evidence against the null model (Kass and Raftery, 1995).

A phylogenetic network was computed with the 38 nearly full-length EV-D68 genome sequences (Supplementary Figure S1). A Neighbor-Net analysis and 1000 bootstrap replicates were performed in SplitsTree 4.13.1 version (Bryant and Moulton, 2004; Huson and Bryant, 2006). The phi test analyzing probability of statistically significant recombination was also performed in SplitsTree.

### Nucleotide Sequence and Accession Numbers

Sequences obtained in this study have been submitted to NCBI’s GenBank sequence database and have been assigned accession numbers KT835373–KT835406 for partial VP1 gene sequences and KT835407–KT835408 for WGS.

### Ethics Statement

Specimens were collected from patients by submitters and sent to PHOL for testing as part of routine clinical service. These data are also used for routine laboratory surveillance, which is a mandate of Public Health Ontario. Therefore, consultation with our organization’s privacy office or ethics committee was not required. To protect patient privacy and confidentiality, data are reported in an anonymized format.

## Results

### Phylogenetic Analysis of VP1 Genes of EV-D68

Phylogenetic analysis with set VP1-All revealed the presence of three distinct clades, A, B, and C, with supporting bootstrap values of 86, 100 and 90%, respectively. Nucleotide and amino acid identity within each clade was 88.9–100% and 88.4–100% for clade A, 89.3–100% and 92.9–100% for clade B, and 94.1–100% and 98.2–100% for clade C, respectively. EV-D68 strains collected in Ontario during the 2014 outbreak belonged to sub-clades B2 (*n* = 31), B1 (*n* = 2), and A2 (*n* = 1) indicating the circulation of multiple sub-clades in Ontario during the same period. The majority of Ontario strains clustered together with USA strains from the same 2014 EV-D68 outbreak and fall into clade B (33/34; 97%) and more specifically, sub-clade B2 (31/34; 91%).

**FIGURE 1:**
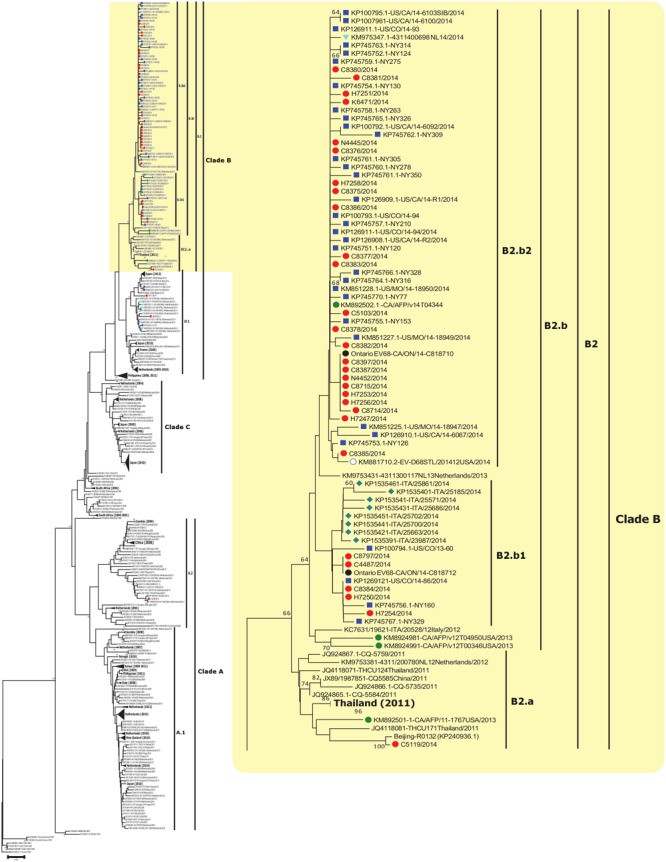
**Phylogenetic tree of EV-D68 detected globally and from Ontario, Canada.** The phylogenetic tree based on the alignment of partial VP1 sequences (339 bp) of EV-D68 was inferred using the Neighbor-Joining method, evolutionary distances were calculated with the Maximum Composite Likelihood method (MCL), and 1,000 Bootstrap replicates were computed to estimate the accuracy of the phylogenetic inference using MEGA v6.06 (http://www.megasoftware.net). Ontario sequences from this study are identified with red filled circles. Sequences collected in 2014 from the USA, Italy, and Netherlands are marked with a blue square, green diamond, and light blue triangle, respectively. AFP sequences collected in 2013 obtained from GenBank are labeled with a green circle. Bootstrap values > 60% are shown next to the nodes. Branch lengths are drawn to the indicated scale, proportion of nucleotide substitutions per site. Some sub-clades are collapsed and labeled in bold to assist with visualization of other clades/sub-clades.

The first EV-D68 sub-clade B2 strains reported globally were from China and the Philippines in 2011. Sub-clade B1 contained the two strains from Ontario and three USA strains from the 2014 outbreak. A distinct cluster was identified within sub-clade B1, consisting of 2014 strains from The Netherlands and 2012 strains from Spain. The global sub-clade B2 sequences subdivide into two distinct branches compiling clusters hereafter referred to as B2.a (containing one strain from Ontario) and B2.b, with the latter documented during the recent 2014 outbreak. Sub-cluster B2.b2 contained the majority (24/34; 71%) of strains from Ontario and USA (31/38; 82%) from the 2014 outbreak (**Figure 1**). Strains within sub-cluster B2.b2 showed nucleotide homology of 98.5–100%. Nucleotide identity of 97.3–99.7% was obtained when B2.b2 were compared to the prototype Fermon strain. Compared to the prototype Fermon strain, all Ontario clade B sequences displayed the following substitutions: N642D, A644T, S647A, G649Q, T650A, and H651D in the BC loop, and N694S, N695S, D696N, S697N, and M700V in DE loop region of VP1 (numbered from the first methionine of the polyprotein gene). Our analysis showed that all strains in sub-cluster B2.b2 responsible for the outbreak in Ontario are distinguished by two signature substitutions, T650A (within the BC loop) and M700V (within the DE loop). Interestingly, among the four available VP1 sequences of strains detected in patients with AFP from the USA in NCBI’s GenBank sequence database, only one collected in 2014 (KM892502.1) clustered with strains of the 2014 outbreak from USA and Ontario, within the main sub-cluster B2.b2.

### Global Distribution of EV-D68 Lineages in Time

**FIGURE 2:**
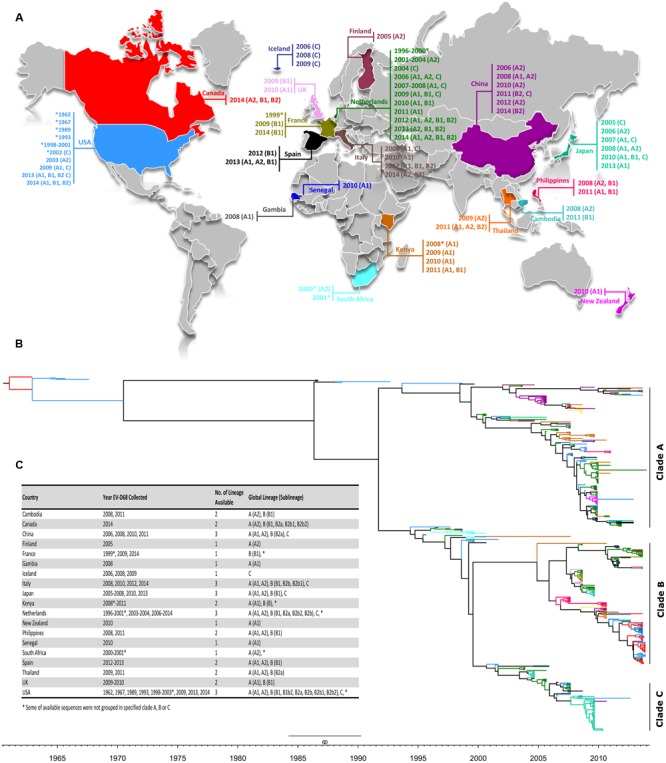
**(A)** Global distribution of EV-D68 as of December 2014. The location and corresponding year of specimen collection for EV-D68 VP1 sequences were obtained from the NCBI GenBank sequence database and color coded by country. EV-D68 clades and sub-clades documented in each country were assigned based on phylogenetic analysis of available partial (339 bp) VP1 nucleotide sequences as shown in **Figure 1**. Strains collected during 2014 are marked for Ontario (red), USA (blue), Netherlands (cyan), and Italy (green). The editable vector map of the world template was downloaded (http://www.presentationmagazine.com/world16maps-vector-editable-507.htm) and edited using PowerPoint and Adobe Photoshop. **(B)** The maximum clade credibility (MCC) tree of VP1 sequences of available strains including the ones from Ontario visualized by FigTree. Branches are color coded according to the world map in **(A)**. Temporal *x*-axis scale represents the sampling dates. **(C)** Global distribution of clades, clusters and sub-clusters.

Using available sequences in GenBank (as of December 16, 2014), the global distribution of EV-D68 along with its period of detection, genotypes, clades and sub-clades were mapped and color coded on a MCC tree according to the place of isolation (**Figures 2A,B**). Lineages of strains with available VP1 sequences from 19 countries (Cambodia, Canada, China, Finland, France, Gambia, Iceland, Italy, Japan, Kenya, Netherlands, New Zealand, the Philippines, Senegal, South Africa, Spain, Thailand, UK, and USA) representing four continents and spanning 53 years (1962–2014) were studied (**Figure 2C**). Five (China, Italy, Japan, Netherlands, and USA) of the 19 countries spanning three continents reported the detection of all three EV-D68 clades (A, B, and C). The number of deposited sequences per country were higher among these five countries than all other countries (*n* = 22–123), except Canada.

### Phylogenetic Analysis of Whole Genome Sequences of EV-D68

**FIGURE 3:**
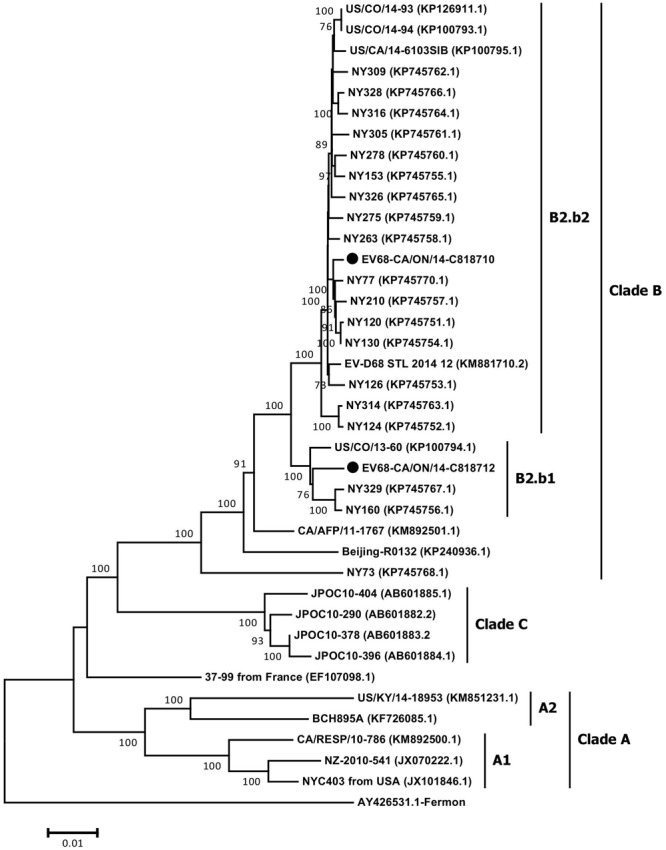
**The phylogenetic relationships of the complete EV-D68 polyprotein gene (6567 nt) of Ontario strains (*n* = 2) to those available in NCBI’s GenBank sequence database (*n* = 36).** Topology was constructed using the Neighbor-Joining analysis and the evolutionary distances were computed using the Maximum Composite Likelihood method available in MEGA v6.06. Bootstrap values (>70%) are shown as percentages derived from 1,000 samplings at the nodes of the tree. The scale bar represents the number of nucleotide substitutions per site. Strains from Ontario are marked with solid black circles.

**FIGURE 4:**
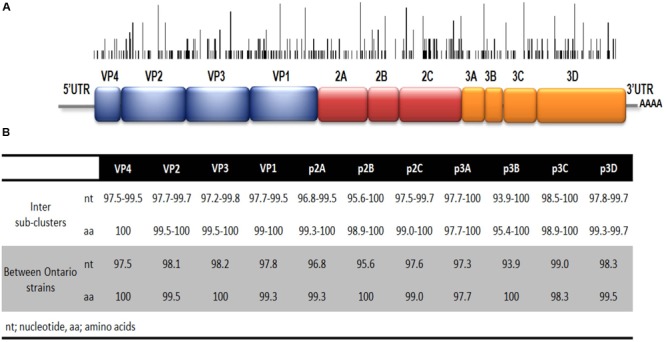
**(A)** Schematic representation of EV-D68 genome. The black bars represent the location of nucleotide variation within strains of clade B2.b2. Height represents number of strains with specific nucleotide variation shown. **(B)** Nucleotide and amino acid identities between sub-clusters B2.b1 and B2.b2 (white area) and between the complete genomes of two strains from Ontario belonging to sub-clades B2.b1 and B2.b2 (gray area).

The full-length genome sequences of two Ontario specimens in the present study were aligned with 36 genome sequences of EV-D68 available from NCBI’s GenBank sequence database. When comparing the WGS of the two Ontario strains representing the two different sub-clusters, B2.b1 and B2.b2, they shared substantial homology of 97.9 and 99.3% in nucleotide and amino acid sequence of the complete polyprotein gene, respectively. Several substitutions were identified within cluster B2.b and between the members of sub-clusters B2.b1 and B2.b2 including I746M, N770S (VP1 protein gene), A844T (p2A gene), S1166N, S1379P (p2C protein gene), I1148V, L1491I (p3A protein gene), D1594E and R1604K (p3D polymerase). Additional mutations were also observed between the two Ontario strains including S112P (VP2), N1142T (p2C), and K1582R (p3C gene). Phylogenetic analysis of WGS data showed the distribution of the three major clades of A, B, and C, with the two Ontario strains belonging to clade B (**Figure 3**). These two Ontario strains showed high identity to the strains isolated from the USA during the 2014 outbreak with nucleotide and amino acid sequences identities of 97.8–99.6 and 99.3–99.9%, respectively (**Figure 4**).

By using NCBI BLAST, isolate NY130 (NCBI accession number KP745754.1), from the USA, and collected in September 2014, was found to be the closest strain to Ontario’s strains in sub-clade B2.b2. Nucleotide sequence identity of 99.7% was obtained between EV68/Ontario/C818710/2014 (NCBI accession number: KT835407.1) and NY130. Nucleotide intra sub-clade identity of 98.9 and 97.8% was observed with the sub-clade B2.b1 and B2.b2 Ontario strains, respectively. Global strains representing each of the two distinct sub-clades (B2.b1 and B2.b2) documented during the 2014 outbreak showed highest and lowest nucleotide identity of 99 and 93.9% within the p3C and p2B regions, respectively.

The phylogenetic network constructed by using a nearly full-length EV-D68 sequences support the clustering of lineages previously observed in the phylogenetic tree (Supplementary Figure S1) and do not display edge (or split) supported by bootstraps near the origin or within the sub-clade B2. Also, the phi test did not show evidence of recombination (*p* = 0.72). These findings suggest that the emergence of sub-lineage B2 is the result of gradual acquisition of mutations rather than more complex models of evolution such as recombination.

### The Phylodynamic History of EV-D68 Genotype Using VP1 Gene and WGS Data

The mean global estimates of evolutionary rates (substitution/site/year), and tMRCA derived from constant and BSP demographic models were implemented in BEAST analyses for all three data sets, VP1-All (*n* = 469), VP1-B2b (*n* = 78), and WGS (*n* = 38). As shown in Supplementary Table S2, the Bayes factor strongly favors the strict molecular clock over the relaxed molecular clock model with both WGS sets (Bayes factor = 900.469) and VP1-All set (Bayes factor = 455.967), indicating that EV-D68 viral strains may have evolved at similar rates. The Bayes factor (2.76) for VP1-B2b set strongly favors a relaxed molecular clock over a strict clock, indicating that different strains may have evolved at significantly different rates (Supplementary Table S2). Similarly, Bayes factors strongly supported the BSP demographic model for each of WGS set, VP1-All set and VP1-B2b set (Supplementary Table S3).

**Table 1** provides estimated mean tMRCA and evolutionary rates of EV-D68 based on the models that are strongly supported by Bayes factor. The mean tMRCA estimates with sets WGS [1960.42 (95% BCI 1957.67–1961.39)] and VP1-All [1961.79 (95% BCI 1960.76–1962.67)] overlap. Similar analysis with the VP1-B2b set estimated the mean tMRCA to be 2012.60 (95% BCI 2011.84–2013.11). The mean rate of EV-D68 evolution using the VP1-All set is estimated to be 4.98 × 10^-3^ substitution/site/year (95% BCI 4.52 × 10^-3^ to 5.45 × 10^-3^). Using the WGS set; the mean evolutionary rate is estimated to be 2.99 × 10^-3^ substitutions/site/year (95% BCI 2.61 × 10^-3^ to 3.30 × 10^-3^). The rate of evolution estimated for the recent EV-D68 emerging strains (with VP1-B2b set) is 9.75 × 10^-3^ substitution/site/year (95% BCI 4.11 × 10^-3^ to 0.016) (**Table 1**).

**Table 1:** Whole genomic and VP1 gene based estimates of mean evolutionary history rates, and tMRCA for Enterovirus D68.

		Mean evolutionary history rates (×10^-3^ substitutions/site/year)			Root height (tMRCA in year)			
		95% BCI			95% BCI		
Datasets	Coalescent method (clock)	Mean	Lower	Upper	Mean decimal year (mean date)	Lower	Upper	First time detected (country)
Set WGS	Bayesian Skyline plot (SC)	2.99	2.61	3.3	(June 2, 1960)	(September 2,1957)	(May 23, 1961)	1962 (USA)
VP1-All	Bayesian Skyline plot (SC)	4.98	4.52	5.45	(October 16, 1961)	(October 5, 1960)	(September 2, 1962)	1963 (USA)
VPl-B2b	Constant size (RC)	9.75	4.11	0.016	(September 5, 2012)	(November 3, 2011)	(February 10, 2013)	2013 (USA)

## Discussion

Historically, EV-D68 virus has been detected sporadically, hence was previously given less priority in routine surveillance by national and international public health agencies. Mapping of clades and sub-clades of EV-D68 virus from different outbreaks from 1962 to 2014 demonstrated the infrequent occurrence and spread of the virus (**Figure 2**). However, all three clades of EV-D68 have been reported in China, Italy, Japan, Netherlands, and the USA (**Figure 2**).

The recently emerged 2014 EV-D68 virus, belonging to cluster B2.b of clade B (**Figure 1**), was widely spread and caused severe respiratory illness across the USA and Canada, disproportionately affecting persons with asthma. The phylogenetic analyses demonstrated that strains from Ontario and the USA grouped in cluster B2.b with the majority belonging to a distinct sub-cluster B2.b2, suggesting a very close relationship between the two adjacent outbreaks. The tree topology suggests that there could have been multiple introductions of strains within B2.b cluster to Ontario from the USA. Notably, all strains from Ontario and USA in this emerging B2.b2 sub-cluster contained two signature substitutions, T650A in the BC loop and M700V in the DE loop of VP1 gene (correspond to positions 98 and 148 of the VP1 coding region) as previously reported among strains circulating in the Philippines during 2013–2014 (Furuse et al., 2015). Further studies involving neutralization and hemagglutination inhibition properties of recent strains along with animal infection models are needed to determine if these mutations play any role in transmissibility, antigenicity, or virulence of the virus. Analysis of nucleotide diversity within the complete polyprotein gene of strains from USA and Ontario revealed that the p2C and p3C protein genes were the most diverse coding regions of the genome, carrying three amino acid substitutions.

The high VP1 sequence homology between strains of B2.a and B2.b, and overlap of the estimated tMRCA of B2.b strains (i.e., 2011–2013) with the detection time of B2.a strains (i.e., 2011–2013 from China, Italy, Netherlands, Thailand, and AFP strains from USA) may suggest emergence of B2.b sub-clade either from or parallel to the B2.a strains. Similar observations were noticed in the emergence of highly pathogenic H5N1 and low pathogenic H5N1 viruses (Monne et al., 2014). A study from Malaysia suggested that the North American EV-D68 outbreak strain was probably descended from lineages of Thailand and Malaysia (Ng et al., 2015).

Both Neighbor-Joining and Bayesian analysis of partial VP1 gene sequences (*n* = 469) from different outbreaks including from Ontario identified three distinct phylogenetic clades (A, B, and C), as previously reported (Tokarz et al., 2012). Genome-scale phylogenetic analysis of the available EV-D68 WGS (including strains from Ontario) showed similar tree topology to that seen for the VP1 gene and supported the existence of the same three clades. Furthermore, the reconstructed MCC tree with set VP1-All showed similar NJ tree topology. Such similarity indicates the selection of a correct and unbiased model in our study, and further emphasizes the discriminatory power of VP1 gene region as a marker in outbreak investigation (**Figures 1**, **2B**). The phylodynamic analyses with sets WGS and VP1-All estimated similar tMRCAs, indicating that the EV-D68 serotype possibly emerged during 1960–1961 or 1961–1962. These estimates correlated with the previous estimates (Tokarz et al., 2012), and are also closely in agreement with the time of first isolation of EV-D68 (Fermon strain) in 1962 in the USA (Schieble et al., 1967). In addition, tMRCA analysis with set VP1-B2b determined that the EV-D68-B2b cluster (which contains the 2014 outbreak EV-D68 strains) as shown in **Figures 1**, **2** possibly emerged during the 2012–2013 season.

Our Bayesian MCMC analyses with three different datasets allowed us to compare the mean estimates of evolution (substitution/site/year) of EV-D68. The mean evolutionary rate of EV-D68 with VP1-All set (∼4.98 × 10^-3^ substitution/site/year) is higher than that of Set WGS (∼2.99 × 10^-3^ substitutions/site/year). The higher estimate of evolutionary rate with the VP1-All set can be attributed to its evolutionary susceptibility, as it contains the antigenic epitopes (Imamura et al., 2014). Similar differences in evolutionary rate were also reported by Tan et al. (2013) and Duvvuri et al. (2015), when they utilized the different length G-gene sequences, and whole genomes of respiratory syncytial virus. Our mean evolutionary estimate from VP1-All set [∼4.98 × 10^-3^ substitution/site/year (95% BCI 4.5–5.5) with a strict clock] is at the lower bound when compared with the estimates of Tokarz et al. (2012) [∼5.8 × 10^-3^ substitution/site/year (95% BCI, 5.2–6.4) with a strict clock and ∼6.2 × 10^-3^ substitution/site/year (95% BCI, 5.4–7.1) with a relaxed clock]. This variation could be explained by the different number and length of sequences utilized in the two analyses (Monne et al., 2014), *n* = 210 with 928 bp; this study, *n* = 469 with 339 bp. The mean rate of evolution with VP1-B2b set (*n* = 74) is ∼9.75 × 10^-3^ substitution/site/year (95% BCI 4.11 × 10^-3^ to 1.6 × 10^-2^), however, the wider credible intervals indicate uncertainty due to limited sequence availability. Other enteroviruses have been associated with respiratory illnesses. For example, enterovirus C117, first identified in a child with pneumonia in Lithuania in 2012, has been found in different countries since and might be associated with respiratory tract infections (Xiang et al., 2014; Van Leer-Buter et al., 2016). Also, human enterovirus 71 (EV-71) is a known etiological agent of hand, foot, and mouth disease and associated with neurological complications. This serotype likely emerged in 1941 and displays substitution rate estimates ranging from 4.2 to 4.6 × 10^-3^/site/year, which is similar to EV-D68 (Tee et al., 2010).

The majority of commercially available molecular assays do not reliably distinguish between enterovirus and rhinovirus, both members of the *Enterovirus* genus. The lack of serotyping of enteroviruses in Canada prior to this outbreak resulted in an unknown prevalence and impact of EV-D68 on the population in the years prior to the outbreak. A Canadian study (2007–2009) observed that acute respiratory infection caused by the enterovirus-rhinovirus group had a more severe clinical course compared to other viruses (Asner et al., 2014). It is unclear if a proportion of those infections could have been caused by EV-D68, as molecular serotyping was not performed in that study. Molecular serotyping of previously collected respiratory samples would contribute to better understanding the epidemiology of EV-D68 in Canada (Granados et al., 2015). The impact of the 2014 EV-D68 North American outbreak emphasizes the need for surveillance of respiratory viruses worldwide. This unexpected event underscores the need for robust molecular serotyping surveillance of enteroviruses, enabling improved serotype specific understanding of virus circulation and disease burden.

## Author Contributions

AE, VD, SP, and JG conceived and designed the study. AE, AL, AP, and PB designed and performed the experiments. AE, VD, and SI analyzed the data, AE carried out the genome characterization and phylogenetic analysis and VD carried out analyses and interpretation of Bayesian phylodynamics. AE, VD, and SI wrote the manuscript. JG, and SP edited the manuscript. All authors reviewed and accepted the final version of manuscript.

## Conflict of Interest Statement

JG has received research grants from GlaxoSmithKline Inc. and Hoffmann-La Roche Ltd to study antiviral resistance in influenza, and from Pfizer Inc. to conduct microbiological surveillance of *Streptococcus pneumoniae*. These activities are not relevant to this study.

The other authors declare that the research was conducted in the absence of any commercial or financial relationships that could be construed as a potential conflict of interest.

## Footnotes

1 http://tree.bio.ed.ac.uk/software/figtree/
