# Supplementary material for: Global Distribution and Evolutionary History of Enterovirus D68, with Emphasis on the 2014 Outbreak in Ontario, Canada
Source: Front Microbiol. 2017 Mar 1;8:257. doi: 10.3389/fmicb.2017.00257 (PMC5331033; doi:10.3389/fmicb.2017.00257)
Supplement: Supplementary file 1 [file Data_Sheet_1.docx]

**Genome comparison and evolutionary history of Enterovirus D68 during the 2014 outbreak in Ontario, Canada**

*Authors*

Alireza Eshaghi^1^†, Venkata R. Duvvuri^1^†, Sandra Isabel^4^, Philip Banh^1^, Aimin Li^1^, Adriana Peci^1^, Samir N. Patel^1,3^, Jonathan B. Gubbay^1,2, 3, 4*^

† These authors contributed equally to this work

*Affiliations*

^1^ Department of Clinical Laboratory and Microbiology Sciences, Public Health Ontario, Toronto, ON, Canada

^2^ Department of Paediatrics, The Hospital for Sick Children, University of Toronto, Toronto, ON, Canada

^3^ Department of Laboratory Medicine and Pathobiology, University of Toronto, Toronto, ON, Canada

^4^ Department of Microbiology, Mount Sinai Hospital, Toronto, ON, Canada

---------

Table S1. Global distribution and years of collection of EV-D68 sequences used in this study

Table S2. Bayes factor between strict (SC) and relaxed (RC) molecular clocks

Table S3: Bayes factor between different coalescent models (Constant population size and Bayesian Skyline plot)

Figure S1. Neighbour-Net

**Table S1.** Global distribution and years of collection of EV-D68 sequences used in this study
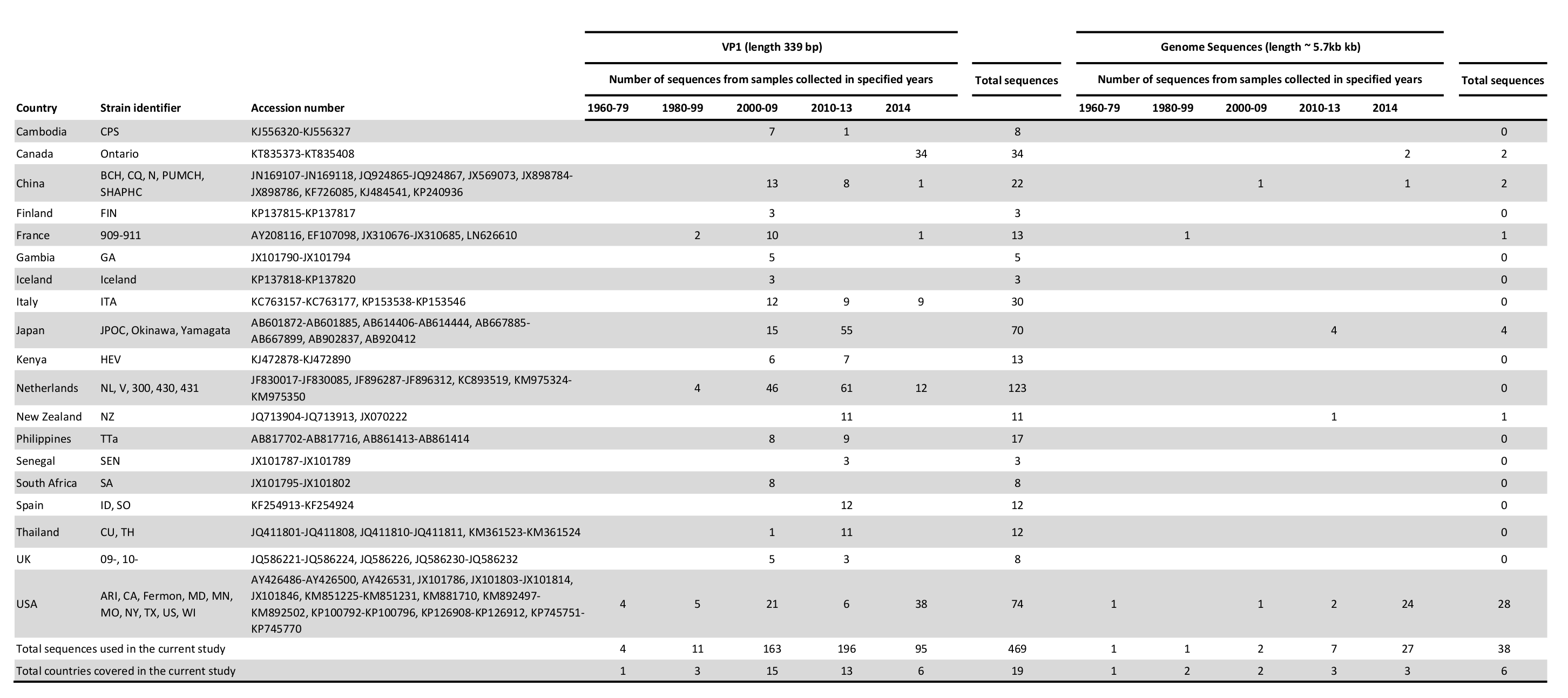


**Table S2.** Bayes factor between strict (SC) and relaxed (RC) molecular clocks


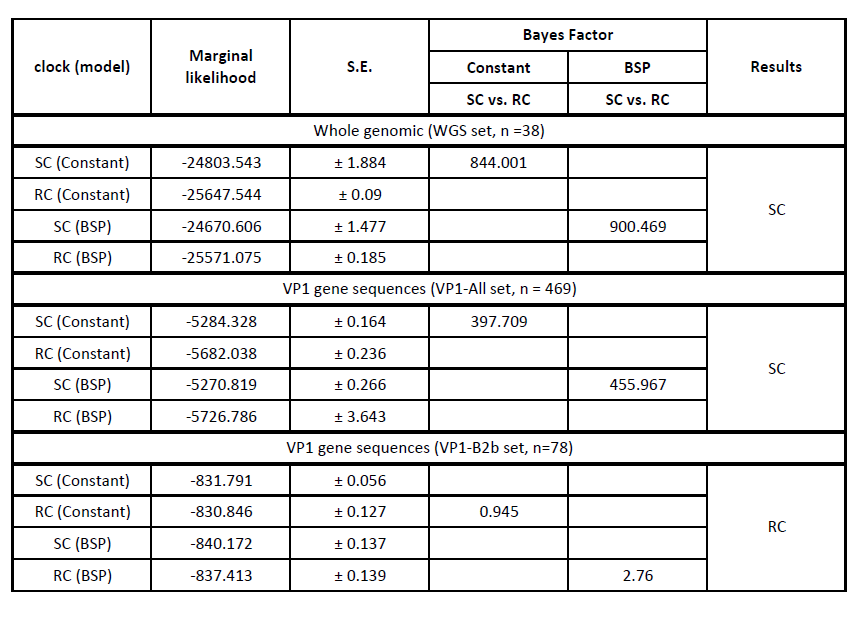


SC: Strict clock, RC: Relaxed clock, BSP: Bayesian skyline plot, S.E: Standard Error

2 > Bayes Factor ≤ 6 indicates positive evidence against the null model; >6 > Bayes Factor ≤ 10 indicates strong evidence against the null model; and Bayes Factor > 10 indicates very strong evidence against the null model

**Table S3.** Bayes factor between different coalescent models (Constant population size and Bayesian Skyline plot)


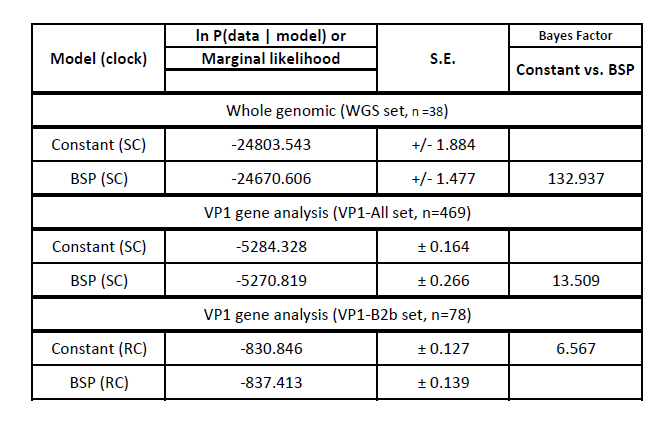


SC: Strict clock, RC: Relaxed clock, BSP: Bayesian skyline plot, S.E: Standard Error


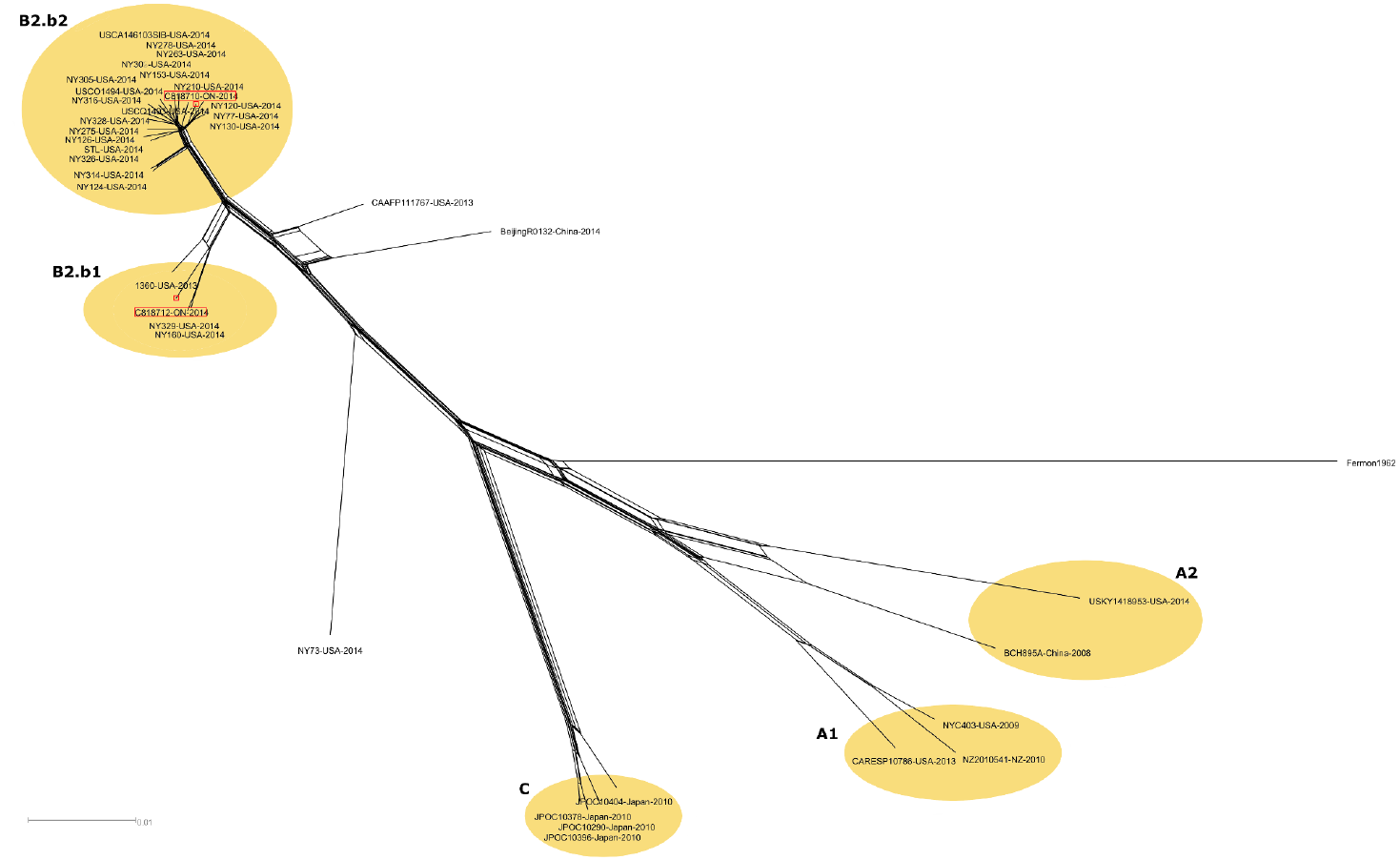


**Figure S1.** Phylogenetic network of complete EV-D68 polyprotein gene (6567 nt) of Ontario strains (*n* = 2) and available in NCBI’s GenBank sequence database (*n* = 36). The Neighbor-Net graph was computed using SplitsTree 4.13.1. The scale bar represents the number of nucleotide substitutions per site. Ontario strains were highlighted in red.
